# From Neurological Severity to Anatomical Burden: An Integrated Clinical–CT Model for Predicting In-Hospital Mortality in Adults with Penetrating Cranial Gunshot Injuries

**DOI:** 10.3390/diagnostics16081246

**Published:** 2026-04-21

**Authors:** Mustafa Emre Sarac, Zeki Boga, Ali Arslan, Ümit Kara, Mehmet Ozer, Ali Harmanoğullarından, Yurdal Gezercan

**Affiliations:** 1 Department of Neurosurgery, Adana City Training and Research Hospital, Adana 01230, Turkey; zekiboga2013@gmail.com (Z.B.); aliarslan26062006@hotmail.com (A.A.); aliharmanoglu@gmail.com (A.H.); gezercan@hotmail.com (Y.G.); 2 Department of Anesthesiology, Adana City Training and Research Hospital, Adana 01230, Turkey; doctorumit@gmail.com; 3 Department of Neurosurgery, Niğde Training and Research Hospital, Niğde 51100, Turkey; mehmetozerdr@gmail.com

**Keywords:** wounds, gunshot, brain injuries, traumatic, Glasgow Coma Scale, tomography, X-ray computed, mortality, risk assessment, prognosis

## Abstract

**Background/Objectives:** Cranial gunshot injuries represent severe traumatic brain injuries associated with high mortality rates. This study investigated whether integrating clinical findings at admission, including GCS score and pupillary response, with a CT-derived anatomical burden score and midline shift improves the prediction of in-hospital mortality. **Methods:** Adult patients aged 18 years and older with penetrating cranial gunshot injuries (*n* = 143) treated at a tertiary referral centre between 1 January 2005 and 31 December 2025 were retrospectively analysed using a single-centre cohort design. All included patients completed in-hospital follow-up, defined as hospital discharge or in-hospital death. Clinical variables, the anatomical burden score, and midline shift were evaluated using a multivariable logistic regression model where the primary outcome was in-hospital mortality. Model performance was assessed using ROC analysis, calibration measures, and bootstrap internal validation. **Results:** The in-hospital mortality rate was 56.6%, with early mortality occurring in 33.6% of patients. In the multivariable analysis, a low admission GCS score (≤8), bilateral non-reactive pupils, an increased anatomical burden score, and midline shift were independently associated with a higher risk of mortality. The model demonstrated good discrimination (AUC = 0.87; 95% CI 0.81–0.93), and similar performance was maintained following internal validation (optimism-corrected AUC = 0.86). The addition of radiological parameters to clinical variables improved model discrimination (ΔAUC = 0.07; 95% CI 0.02–0.11). **Conclusions:** The combined evaluation of admission clinical findings and CT-based anatomical parameters may support a more structured early estimation of in-hospital mortality risk in adult patients with penetrating cranial gunshot injuries.

## 1. Introduction

Cranial gunshot injuries (CGSIs) constitute a major public health concern in civilian settings and represent one of the most devastating forms of penetrating traumatic brain injuries (TBIs) frequently encountered in emergency neurosurgical practice [1,2]. As one of the most severe forms of TBIs, these injuries are associated with reported mortality rates ranging from approximately 40% to 60% across civilian series [3]. Frequently concentrated among young male individuals, these injuries are clinically significant due to their high early mortality rates. This elevated mortality indicates the vital role of early-phase risk stratification in clinical decision-making [2].

In CGSIs, while ballistic characteristics provide important information regarding the mechanism of injury, they are insufficient on their own to fully explain clinical outcomes, as they do not account for factors such as the extent of intracranial anatomical burden and the degree of involvement of critical anatomical structures, which are crucial for understanding prognosis [4]. Mortality risk should be evaluated by considering the extent of intracranial anatomical burden and the degree of involvement of critical anatomical structures, together with the neurological status at admission [3]. In both paediatric and adult populations, neurological status at admission is closely associated with prognosis, with mortality increasing markedly in patients presenting with a severe neurological presentation [5].

In particular, patients with a GCS score ≤ 8 at admission exhibit a significantly higher risk of mortality. For this reason, the GCS is regarded as one of the most robust and practical indicators of clinical severity [6]. Nevertheless, outcomes are influenced not only by clinical parameters but also by the anatomical distribution of affected brain regions. The literature reports that multilobar involvement, a cross-midline projectile trajectory, and injury to deep structures are associated with poor prognosis [5,7]. Posterior fossa injuries, in particular, may follow a rapidly fatal course in the early phase due to the limited compartmental volume and proximity to the brainstem and are frequently reported as independent predictors of mortality [8,9]. Non-contrast computed tomography (CT) plays a fundamental role in early risk assessment by delineating the anatomical distribution of injuries [10].

However, radiological parameters have largely been analysed as isolated variables in the existing literature, and integrated approaches that capture the overall intracranial anatomical burden remain limited. Studies that combine clinical severity indicators such as the GCS with CT-based anatomical distribution and midline shift within the same multivariable model, and systematically report model performance in terms of both discrimination and calibration, remain relatively scarce [3,5,7]. In this context, summarising the cumulative topographical extent of injury using a structured composite indicator may facilitate a more coherent assessment of radiological injury burden.

In the present study, clinical and radiological variables were jointly evaluated in adult patients with penetrating CGSIs treated at a tertiary neurosurgical referral centre. The independent effects of GCS score at admission and pupillary response, together with CT-based anatomical distribution, on in-hospital mortality were investigated. The aim of this study was to determine whether the combined evaluation of clinical severity with CT-based anatomical burden and midline shift provides incremental value in predicting in-hospital mortality. For this purpose, a pragmatic composite “anatomical burden score” was defined to summarise the cumulative intracranial extent of injury based on CT features previously associated with poor outcomes and analysed together with clinical variables within a multivariable model.

## 2. Materials and Methods

### 2.1. Study Design and Patient Selection

This study is a single-centre retrospective cohort analysis, including adults with CGSIs treated at our institution between 1 January 2005, and 31 December 2025. The study was based on routinely collected clinical and imaging data obtained during standard patient care. All the included patients completed in-hospital follow-up, defined as hospital discharge or in-hospital death, and no patient remained hospitalised at the end of the study period. Our centre is a tertiary neurosurgical referral centre located in a region where CGSIs are frequently encountered.

Initially, all firearm-related injury cases were screened; a total of 149 patients were identified who had an intracranial penetration confirmed by non-contrast brain CT, were aged 18 years or older, and had at least one documented neurological examination recorded in the emergency department. To ensure methodological homogeneity, only penetrating CGSIs with a single-entry wound were included, and perforating injuries with both entry and exit wounds were excluded. Patients who were brought to the emergency department with cardiac arrest or who died before neurosurgical evaluation were also excluded, along with patients who had superficial or tangential injuries not penetrating the skull or widespread fragmentation injuries involving numerous small metallic fragments such as shotgun pellets.

Six patients were excluded due to incomplete clinical or imaging data, resulting in a final analytic cohort of 143 patients. The rate of missing data was 4.0% for the total cohort, and analyses were conducted using complete-case data. The baseline demographic and clinical characteristics of the patients excluded from analysis due to missing data were compared with those of the included patients, and no significant differences were identified.

### 2.2. Imaging and Clinical Assessment

All patients underwent non-contrast cranial computed tomography (CT) at admission in accordance with the standardised TBI management protocol implemented at our institution. The imaging protocol included a tube voltage of 120 kVp, automatic mA modulation, and a 5 mm axial slice thickness. The scanning range extended from the vertex to the foramen magnum. In clinically stable patients, a follow-up CT scan was performed within the first two hours to assess early haematoma progression and the development of mass effect.

Admission CT scans were systematically evaluated for the anatomical location and extent of injury. The following radiological variables were recorded: multilobar involvement, posterior fossa involvement, cross-midline projectile trajectory, intraventricular haemorrhage (IVH), deep structure involvement (basal ganglia, thalamus, or brainstem), and midline shift (in millimetres). Midline shift was measured at the level of the septum pellucidum at the point of maximal deviation. Measurements were obtained using an electronic calliper within the digital imaging system and were recorded in millimetres. Radiological variables were coded as binary variables (present/absent).

Clinical assessment at admission included the Glasgow Coma Scale (GCS), pupillary light reflex, vital parameters, and mechanism of injury. In patients who were intubated upon arrival or required intubation in the emergency department, the GCS was recorded according to established TBI assessment principles, taking into account the potential confounding effect of sedation. In intubated patients, the verbal response component was documented as “T”, and the total GCS score was calculated based on the motor and eye-opening components in accordance with standard trauma literature recommendations. Pupillary response was classified as bilateral reactive, unilateral non-reactive, or bilateral non-reactive. Pupillary assessment was based on the first reliable neurological examination performed prior to sedative administration or during a period of minimal sedation. Clinical variables were analysed based on the earliest reliable neurological findings documented in the emergency department.

### 2.3. Surgical and Medical Treatment Protocol

Treatment strategy was determined based on combined clinical and radiological findings. Surgical decision-making followed the TBI management algorithm implemented at our centre during the study period and was based on the clinical judgement of the responsible senior neurosurgeon. Surgical indications were defined according to pre-established clinical and radiological criteria and were applied independently of the prognostic analysis.

Surgical management was performed in cases of intraparenchymal haematoma causing mass effect, diffuse cerebral oedema associated with midline shift, contaminated open cranial injuries, presence of cerebrospinal fluid (CSF) fistula, and intraparenchymal bone or bullet fragments requiring removal for clinical reasons. Procedures included haematoma evacuation, decompressive craniectomy, foreign body removal where appropriate, dural repair, and wound debridement. Bullet or bone fragments embedded in critical anatomical structures such as the thalamus, brainstem, or basal ganglia were not removed due to the risk of additional neurological injury.

In non-surgical (conservative) management, hyperosmolar therapy was administered to reduce intracranial pressure. Depending on the clinical and haemodynamic response, renal function, and serum sodium levels, either 20% mannitol (loading dose 0.5–1 g/kg; maintenance 0.25–0.5 g/kg) or 3% hypertonic saline (loading dose 2–4 mL/kg; low-rate maintenance infusion according to clinical response) was used [10,11]. The choice of hyperosmolar therapy was individualised according to haemodynamic stability, serum sodium concentration, and comorbidities. Serum osmolality and electrolyte levels were monitored regularly. In all patients, a head elevation of 30–45 degrees was maintained to reduce cerebral oedema [12].

In patients requiring intubation, sedation for neuroprotective purposes was achieved following a propofol (1–2 mg/kg loading; 1–3 mg/kg/h maintenance) or midazolam (0.1–0.2 mg/kg loading; 0.05–0.1 mg/kg/h maintenance) protocol. Sedation depth was titrated according to haemodynamic stability and neurological monitoring requirements [13]. Ceftriaxone was administered at 2 g/day for infection prophylaxis, and phenytoin (15 mg/kg loading followed by 100 mg every 8 h) was given for seizure prophylaxis [10,14]. Ventilation–perfusion management aimed to maintain PaCO_2_ between 35 and 40 mmHg to minimise adverse effects on intracranial pressure [15]. All intensive care management was conducted in a neurosurgical intensive care unit staffed by trained and experienced personnel.

Treatment variables were not included in the primary prognostic model because the model was designed to reflect admission-based risk stratification using variables available at initial presentation. Treatment decisions were made after clinical and radiological assessment and may, therefore, lie on the causal pathway between admission severity and outcome. Inclusion of such post-admission variables in the primary model could introduce bias related to treatment allocation. An additional multivariable model including treatment category was constructed to evaluate the potential impact of treatment-related factors.

### 2.4. Classification of Injury Patterns

Injury patterns were classified according to anatomical location based on admission CT findings as frontal, temporal, parietal, occipital, or posterior fossa involvement. Multilobar involvement was defined as the simultaneous involvement of two or more cerebral lobes.

Admission CT scans were further evaluated for cross-midline projectile trajectory, intraventricular haemorrhage (IVH), deep structure involvement (basal ganglia, thalamus, or brainstem), and midline shift (mm), each recorded as separate radiological variables.

The anatomical burden score was constructed to quantitatively represent the overall extent of intracranial injury based on four predefined radiological parameters. One point was assigned to each of the following components: multilobar involvement, cross-midline trajectory, IVH, and deep structure involvement, resulting in a total score ranging from 0 to 4. These components were selected because they reflect distinct aspects of intracranial injury distribution that have been repeatedly associated with poor outcomes in prior CGSI studies. Equal weighting was deliberately adopted to preserve simplicity and reproducibility within the present retrospective cohort, allowing the score to function as a pragmatic composite indicator of overall injury extent rather than a weighted prognostic index. Posterior fossa involvement was evaluated separately rather than incorporated into the composite score because of its distinct anatomical compartment and potentially different pathophysiological implications.

**Figure 1:**
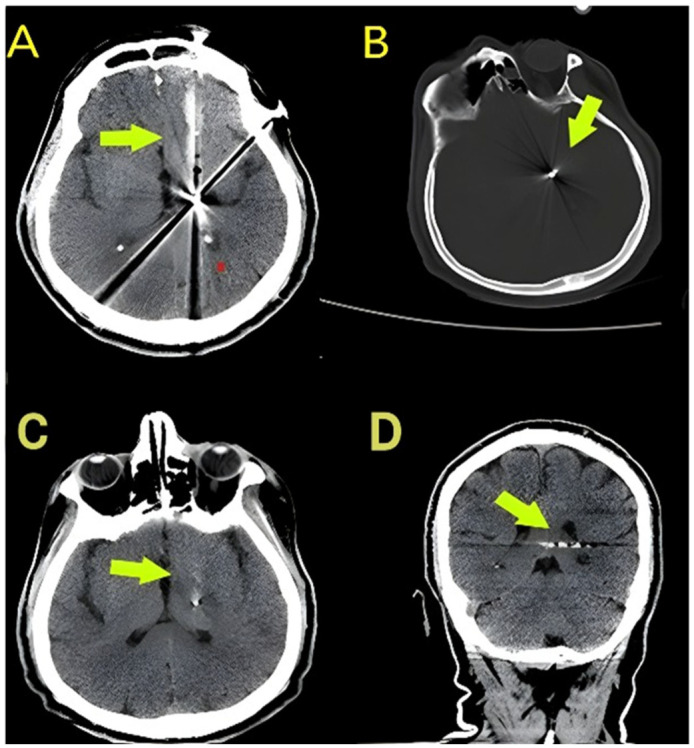
Representative admission CT images demonstrating an anatomical burden score of 1. (**A**) Axial non-contrast CT showing a projectile tract confined to the frontal lobe with associated parenchymal haemorrhage (arrow). (**B**) Axial bone-window CT demonstrating the retained metallic fragment without cross-midline extension (arrow). (**C**) Axial CT image showing deep structure involvement along the projectile tract (arrow). (**D**) Coronal reconstruction confirming absence of intraventricular haemorrhage and no cross-midline trajectory (arrow). The anatomical burden score was 1, reflecting deep structure involvement without multilobar extension, cross-midline trajectory, or intraventricular haemorrhage.

**Figure 2:**
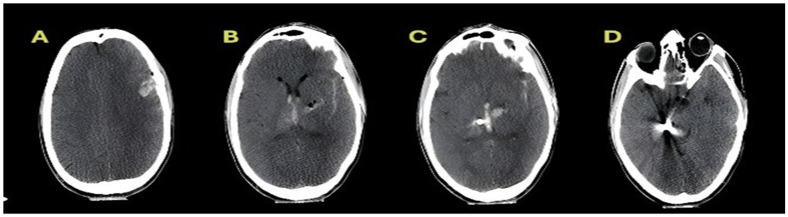
Representative admission CT images demonstrating an anatomical burden score of 3. (**A**) Axial non-contrast CT at the supratentorial level showing a unilateral cortical entry site without multilobar involvement. (**B**) Axial CT demonstrating intraventricular haemorrhage (IVH) with associated deep structure involvement. (**C**) Axial CT slice showing deep structure injury with intraventricular haemorrhage and projectile trajectory crossing the midline. (**D**) Axial CT image illustrating a cross-midline projectile trajectory with deep structure involvement. The anatomical burden score was 3, reflecting a cross-midline trajectory, intraventricular haemorrhage, and deep structural involvement in the absence of multilobar cortical extension.

Representative CT images illustrating the radiological components of the anatomical burden score are shown in Figure 1 and Figure 2. The distribution of scores and mortality rates across categories are presented descriptively; in prognostic analyses, the anatomical burden score was included as a continuous variable. The midline shift was not included in the calculation of anatomical burden score but was analysed separately as a continuous variable.

### 2.5. Data Collection

Data were retrospectively obtained from the hospital’s electronic and written medical records, operative reports, intensive care and outpatient follow-up documentation maintained by the senior neurosurgical consultant, and imaging archives. Clinical data included demographic characteristics, neurological status at admission, pupillary response, treatment approaches, and complications. Radiological variables were recorded from admission CT scans using a pre-structured standard data collection form.

To enhance the accuracy and reliability of radiological classification, admission CT scans were initially evaluated by a neurosurgeon at our institution. For reliability assessment, the scans were independently re-evaluated by a second neurosurgeon from an external institution who was blinded to clinical outcome data. Interobserver agreement analysis was conducted for 40 randomly selected cases from the study cohort. Cohen’s kappa coefficient was calculated for binary variables, and an intraclass correlation coefficient (ICC) based on a two-way random-effects absolute agreement model was calculated for midline shift measurements. The classification was done based on the criteria provided for each operational category. All clinical and radiological information was recorded and summarised, and analyses were performed on the final dataset.

### 2.6. Follow-Up and Clinical Outcomes

The clinical course was retrospectively assessed based on neurological status during hospitalisation, early and late complications, and survival status. Two mortality endpoints were defined: early mortality and in-hospital mortality. Early mortality was defined as death within the first 72 h after admission, while in-hospital mortality included all deaths that occurred during the index hospitalisation. The primary outcome was predefined as in-hospital mortality, and prognostic model analyses were conducted using this endpoint. Early mortality was evaluated as a secondary descriptive endpoint. No multivariable modelling was performed for early mortality, as this analysis was conducted solely to assess the directional consistency of the variables.

Survival status was verified using hospital electronic records, the national electronic patient registry, and the national death notification system. In cases of conflicting information, the most recent official death record was considered definitive. For patients transferred to external centres, the last confirmed recording date was used.

Functional outcomes were classified according to the Glasgow Outcome Scale (GOS) based on neurological status at discharge. GOS classification was derived from discharge summaries and neurological examination records. Functional outcomes were analysed descriptively, and they were not included in the multivariable mortality prediction model.

### 2.7. Definition of Complications

Complications were defined according to pre-established operational criteria based on clinical documentation and imaging findings. Meningitis was defined as the presence of fever and meningeal irritation signs accompanied by CSF findings suggestive of infection based on biochemical and microbiological analysis, or the initiation of targeted antibiotic therapy based on clinical suspicion. Pulmonary infection was defined as new or progressive radiological pulmonary infiltrates accompanied by clinical signs of infection (fever, leukocytosis, purulent secretions, etc.) requiring antibiotic therapy. CSF fistula was defined as clinical evidence of rhinorrhoea or otorrhoea and confirmation of CSF leakage through clinical and imaging assessment.

Only complications occurring during hospitalisation were recorded, and evaluation was limited to patients undergoing active inpatient follow-up. The timing of complications was verified through daily clinical notes and imaging records.

### 2.8. Statistical Analysis

Statistical analyses were performed using IBM SPSS Statistics (version 22.0; IBM Corp., Armonk, NY, USA) and R software (version 4.3.2; R Foundation for Statistical Computing, Vienna, Austria). The distribution of continuous variables was assessed using visual methods (histograms and Q–Q plots) together with the Shapiro–Wilk test. Continuous variables are presented as mean ± standard deviation (SD) when normally distributed and as median with interquartile range (IQR) when non-normally distributed. Categorical variables are presented as counts and percentages. Comparisons between groups were performed using the chi-square test or Fisher’s exact test for categorical variables. Continuous variables were compared using Student’s *t*-test when normally distributed and the Mann–Whitney U test when non-normally distributed.

Univariable logistic regression analyses were performed to evaluate the association between candidate predictors and in-hospital mortality. Candidate predictors considered for modelling included age, the GCS admission category, pupillary responses, posterior fossa involvement, anatomical burden scores, and midline shifts, all of which were selected based on clinical relevance and availability at admission. Variables were not selected using automated stepwise procedures; rather, model construction was guided by clinical relevance and the objective of developing an admission-based prognostic model. Variables showing a univariable association (*p* < 0.10) were considered for inclusion; however, the final model was defined with emphasis on clinical relevance and parsimony. Odds ratios (ORs) with 95% confidence intervals (CIs) are reported for all regression analyses. A multivariable logistic regression model was constructed for the primary endpoint of in-hospital mortality. Model complexity was restricted to maintain an adequate events-per-variable (EPV) ratio (≥10) in order to reduce the risk of model overfitting. The anatomical burden score was entered as a continuous variable. In addition, a second multivariable model including treatment category was constructed to assess the potential influence of treatment-related factors on the associations between admission variables and in-hospital mortality.

The assumption of linearity between continuous predictors and the logit was evaluated graphically. Since visual inspection of the predictor–logit plots did not suggest any meaningful deviation from linearity for the continuous predictors included in the model, these variables were retained in the model as continuous terms. Multicollinearity was assessed using the variance inflation factor (VIF), with VIF > 5 considered indicative of potential collinearity. To address potential separation, results were confirmed by Firth’s penalised logistic regression.

Model performance was evaluated in terms of discrimination and calibration. Discrimination was assessed using the receiver operating characteristic (ROC) curve and area under the curve (AUC). Internal validation was conducted using 1000 bootstrap resamples, and optimism-corrected AUC values were calculated. Bootstrap resampling was applied to the final fitted model in order to estimate optimism in the discrimination and calibration metrics. Calibration was assessed using calibration plots together with the calibration slope, intercept, and Brier score. The linear trend across anatomical burden score categories was assessed using the Cochran–Armitage test. No formal a priori sample size calculation was performed; however, adequacy of the sample for multivariable modelling was supported by maintaining an events-per-variable (EPV) ratio ≥ 10 and by internal validation procedures, including bootstrap resampling and penalised regression analysis. A two-sided *p*-value < 0.05 was considered statistically significant for all analyses.

### 2.9. Ethical Approval and Informed Consent

This study was conducted in accordance with the principles of the Declaration of Helsinki and was approved by the Clinical Research Ethics Committee of Adana City Training and Research Hospital (Meeting No.: 23; Decision No.: 1211; Approval Date: 25 February 2026). The study was designed as a retrospective analysis of routinely collected clinical data, and all data had been generated as part of standard clinical care prior to study conception. Data extraction and statistical analyses were performed after ethics approval had been obtained. Because of the retrospective design of the study, the ethics committee waived the requirement for additional study-specific informed consent. Written consent permitting the use of anonymised clinical data for scientific purposes had previously been obtained from patients or their legal representatives in accordance with institutional policy.

## 3. Results

### 3.1. Demographic Characteristics and Clinical Status at Admission

Among the 143 adult patients included in the study, in-hospital mortality occurred in 81 patients (56.6%). Compared with survivors, non-survivors were significantly older and more frequently presented with severe neurological impairment (GCS ≤ 8) and bilateral non-reactive pupils at admission. Higher anatomical burden scores and a greater midline shift were also observed among non-survivors. Baseline demographic, clinical, and radiological characteristics stratified according to in-hospital mortality are presented in Table 1.

### 3.2. Radiological Characteristics and Patterns of Anatomical Injury

Based on non-contrast brain CT performed at admission, multilobar involvement was the most frequently observed injury pattern, identified in 55 patients (38.5%). Posterior fossa involvement was observed in 23 patients (16.1%), a cross-midline projectile trajectory was present in 39 patients (27.3%), intraventricular haemorrhage (IVH) was detected in 40 patients (28.0%), and deep structure involvement was seen in 27 patients (18.9%). The anatomical burden score ranged from 0 to 4, with a distribution as follows: score of 0 in 44 patients (30.8%), score of 1 in 52 patients (36.4%), score of 2 in 37 patients (25.9%), score of 3 in 9 patients (6.3%), and score of 4 in 1 patient (0.7%).

As the anatomical burden score increased, a stepwise increase in in-hospital mortality was observed, and this linear trend was statistically significant (*p* < 0.001; Table 2).

Midline shift had a median value of 5 mm in the entire cohort (IQR 0–8). Among patients who experienced in-hospital mortality, the median midline shift was 8 mm (IQR 4–12), whereas in survivors, it was 2 mm (IQR 0–5), and this difference was statistically significant (Mann–Whitney U, *p* < 0.001).

In the interobserver agreement analysis of radiological variables, Cohen’s kappa coefficient was 0.82 (95% CI 0.70–0.93) for multilobar involvement, 0.76 (95% CI 0.62–0.89) for cross-midline trajectory, 0.79 (95% CI 0.66–0.91) for intraventricular haemorrhage, and 0.74 (95% CI 0.58–0.88) for deep structure involvement. For midline shift measurements, interobserver agreement was assessed using the intraclass correlation coefficient (ICC) based on a two-way random-effects absolute agreement model, demonstrating a high agreement (ICC = 0.91; 95% CI 0.84–0.95).

### 3.3. Distribution of Treatment and Clinical Characteristics at Admission

In the study population, the majority of patients underwent surgical intervention, while a smaller group was managed conservatively. Regarding surgical procedures, decompressive craniectomy with haematoma evacuation was the most frequently performed intervention, followed by more limited surgical procedures (Table 3).

When the treatment groups were examined with respect to clinical characteristics at admission, patients who underwent surgical intervention demonstrated a greater neurological severity. The median GCS score at admission was lower in the surgical group, with the lowest GCS scores observed in the subgroup undergoing decompressive craniectomy.

Although in-hospital mortality rate was higher in the surgical group, the difference should be interpreted in the context of neurological severity at admission and treatment indications. A detailed distribution of treatment groups is presented in Table 3.

### 3.4. Complications

Among the 62 surviving patients, at least one complication developed in 16 cases (25.8%). The most frequent complication was meningitis (*n* = 10, 16.1%), followed by pulmonary infection (*n* = 4, 6.5%) and CSF fistula (*n* = 2, 3.2%).

### 3.5. Mortality, Clinical Outcomes, and Model Performance

The overall in-hospital mortality rate was 56.6% (81/143), with early mortality (within the first 72 h) observed in 33.6% (48/143) of patients. When stratified by GCS scores at admission, in-hospital mortality was 79.5% (66/83) in the GCS ≤ 8 group and 25.0% (15/60) in the GCS > 8 group (*p* < 0.001). Among survivors, functional outcomes differed markedly between groups: in the GCS ≤ 8 group, severe disability was observed in 47.1% (8/17) of patients, moderate disability was observed in 35.3% (6/17), and favourable recovery was seen in 17.6% (3/17). In contrast, in the GCS > 8 group, severe disability occurred in 8.9% (4/45) of patients, moderate disability occurred in 17.8% (8/45), and good recovery occurred in 73.3% (33/45). Percentages for functional outcomes were calculated among survivors in each group.

**Figure 3:**
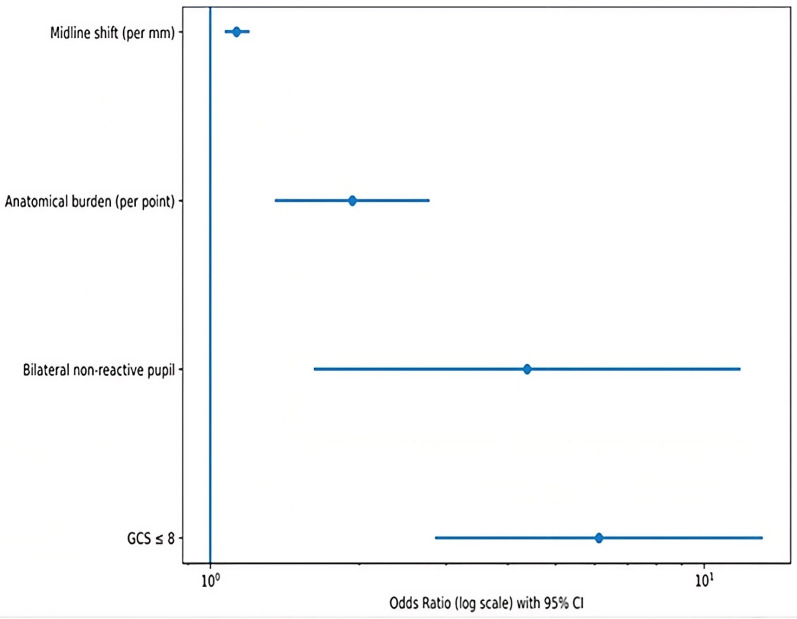
Independent predictors of in-hospital mortality. Forest plot showing odds ratios (ORs) with 95% confidence intervals derived from the final multivariable logistic regression model. Continuous variables were modelled per unit increase. Odds ratios are displayed on a logarithmic scale, and the vertical line indicates OR = 1. Model stability was confirmed using Firth penalised logistic regression.

In the multivariable logistic regression model for in-hospital mortality, midline shift (OR 1.13 per mm; 95% CI 1.07–1.20; *p* < 0.001), anatomical burden score (OR 1.94 per point; 95% CI 1.35–2.78; *p* < 0.001), bilateral non-reactive pupils (OR 4.38; 95% CI 1.62–11.84; *p* = 0.003), and admission GCS ≤ 8 (OR 6.12; 95% CI 2.85–13.15; *p* < 0.001) were independently associated with mortality (Table 4; Figure 3). Age and posterior fossa involvement were associated with mortality in the univariable analysis but did not retain their significance after adjustment in the multivariable model. A second multivariable logistic regression model including treatment category was constructed to evaluate the potential influence of treatment-related factors, and inclusion of treatment resulted in modest attenuation of effect estimates without altering their direction or statistical significance; for example, the odds ratio for admission GCS ≤ 8 was 6.12 (95% CI 2.85–13.15) in the primary model and 5.82 (95% CI 2.60–13.02) after inclusion of treatment, while the treatment variable itself was not significantly associated with in-hospital mortality in the adjusted model (OR 0.89; 95% CI 0.42–1.87; *p* = 0.76), as shown in Table 4.

**Figure 4:**
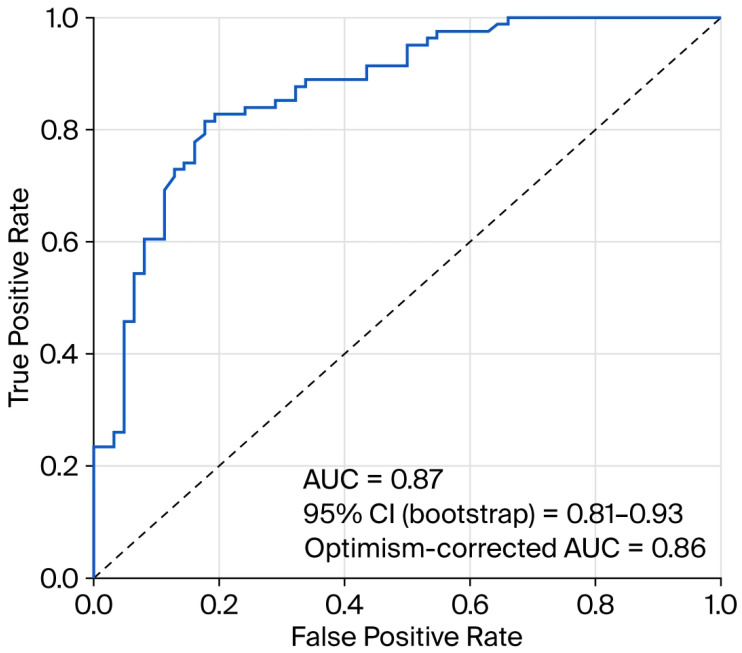
Receiver operating characteristic (ROC) curve of the final multivariable model for in-hospital mortality. Discriminative performance was quantified by the area under the curve (AUC = 0.87). The 95% confidence interval was estimated using bootstrap resampling, and the optimism-corrected AUC was obtained through bootstrap internal validation. The diagonal dashed line represents discrimination at the chance level.

Model discrimination assessed with ROC analysis yielded an AUC of 0.87 (95% CI 0.81–0.93) (Figure 4). After 1000-bootstrap internal validation, the optimism-corrected AUC was 0.86. The clinical baseline model (GCS ≤ 8 and pupillary response) showed an AUC of 0.80, which increased to 0.87 after the addition of the anatomical burden score and midline shift (ΔAUC = 0.07; 95% CI 0.02–0.11) (Figure 4). AUC estimates were comparable after inclusion of treatment variables in the extended model, indicating preserved discriminative performance.

**Figure 5:**
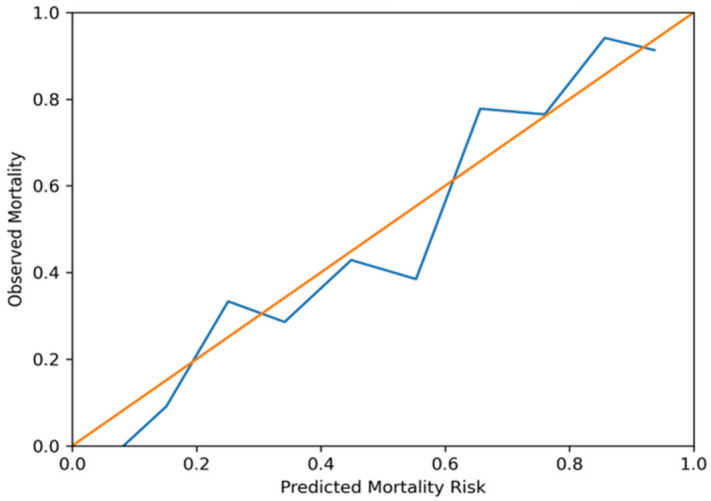
Calibration curve for the in-hospital mortality model. Observed mortality is plotted against predicted probabilities across deciles of predicted risk. The diagonal line represents perfect calibration.

No significant multicollinearity was detected among model variables (all VIF < 2). The assumption of linearity in the logit for continuous variables was graphically assessed, and no meaningful deviation was observed. Calibration analysis demonstrated good agreement between the predicted and observed mortality rates (calibration slope 0.98; intercept −0.03; Brier score 0.18) (Figure 5) and remained stable after inclusion of treatment variables.

## 4. Discussion

In this study of adult patients with CGSIs, four variables were identified as independently associated with in-hospital mortality: low admission GCS score (≤8), bilateral non-reactive pupils, increased anatomical burden score, and greater midline shift. The overall in-hospital mortality rate was 56.6%, and early mortality within the first 72 h occurred in 48 patients (33.6% of the cohort), corresponding to 59.3% of all deaths. This finding indicates that mortality risk is largely determined in the early phase after injury and underscores the early prognostic relevance of clinical and radiological parameters obtained at presentation. The baseline clinical model based on GCS and pupillary response yielded an AUC of 0.80, which increased to 0.87 (ΔAUC = 0.07) after the addition of radiological variables, supporting the incremental prognostic contribution of CT-based indicators beyond the initial clinical severity. The consistent performance of the model in terms of both discrimination and calibration indicates that integrating clinical findings with CT-based anatomical indicators may support structured early risk estimation. In addition, the inclusion of treatment variables in an extended model did not materially alter the magnitude or direction of the associations observed for the primary predictors, indicating that these relationships are robust and not solely driven by treatment-related factors.

Admission GCS score has long been regarded as one of the strongest prognostic determinants in CGSIs. Large civilian series have demonstrated a strong association between a low GCS score and mortality [3,16]. Similarly, multicentre studies involving TBI populations have confirmed the central role of the GCS in outcome prediction [17]. Reports on cohorts in war settings have likewise shown a direct association between neurological severity at presentation and mortality [18]. In our cohort, the GCS remains an independent predictor of mortality, reaffirming its prognostic importance; however, it is insufficient in accounting for the full variability of risk when considered alone.

The prognostic significance of pupillary response is particularly evident in severe brain injury, where a loss of pupillary reactivity is commonly associated with increased intracranial pressure or brainstem dysfunction. In adult civilian series, bilateral non-reactive pupils have shown a strong association with mortality [5,19,20], and paediatric CGSI studies have also reported close associations between pupillary findings, mortality, and functional outcomes [21]. Emami et al. further demonstrated that pupillary parameters significantly influence outcomes in both adult and paediatric severe TBIs [22]. In our study, bilateral non-reactive pupils retained independent significance, supporting the continued value of detailed neurological examination in early prognostication.

Radiological parameters have predominantly been evaluated as isolated variables in the literature. Multilobar involvement, cross-midline projectile trajectory, deep structure injury, intraventricular haemorrhage, and posterior fossa involvement have each been associated with mortality in various cohorts [3,5,7]. However, such an approach may be limited in capturing the overall intracranial injury burden in a comprehensive manner [3]. While posterior fossa involvement in our cohort was associated with mortality in the univariable analysis, it did not retain independent significance in the multivariable model after adjustment for clinical severity and overall anatomical burden. This observation suggests that certain radiological variables may overlap with clinical severity and other anatomical parameters, thereby limiting the interpretability of their isolated effects. Accordingly, an integrated approach capable of representing the total intracranial extent of injury may offer additional prognostic value. In the present study, radiological parameters were integrated into a composite anatomical burden score, allowing the injury extent to be modelled as a quantitative and graded variable. Analysing the anatomical burden score as a continuous predictor enabled an evaluation of the incremental change in mortality risk per unit increase. The significant association between an increasing score and mortality supports the prognostic sensitivity of this integrated approach. Although equal weighting was applied to the components of the anatomical burden score to preserve simplicity and reproducibility, this approach does not imply that each component contributes equally to outcome, and the score should, therefore, be interpreted as a pragmatic representation of cumulative injury burden rather than a weighted prognostic index. The observed improvement in discrimination after the addition of radiological variables, together with preserved calibration, suggests that CT findings can be systematically integrated into a structured risk stratification framework. While established CT-based classification systems such as the Marshall and Rotterdam scores have been widely used in TBI populations, their applicability to penetrating cranial injuries remains limited, and the present approach was intended to provide a complementary framework tailored to the specific characteristics of CGSIs rather than to replace existing scoring systems [23,24].

A midline shift is widely recognised as an objective indicator of mass effects and secondary injuries, and its association with mortality in TBI has been previously demonstrated [25]. While the anatomical burden score reflects the topographical extent of intracranial damage, a midline shift represents the dynamic consequence of intracranial mass effect; thus, the two parameters capture related but complementary pathophysiological dimensions. The minimal change observed in the effect estimate for midline shift after inclusion of treatment variables is consistent with its role as a direct marker of mass effect, which is less influenced by post-admission management decisions.

Evaluating the anatomical burden score as a continuous variable allows us to assess graded changes in mortality risk corresponding to increasing injury extent. This approach facilitates an understanding of how risk evolves as anatomical involvement progresses and, when considered alongside clinical variables, may provide a more nuanced representation of overall disease severity.

Taken together, these findings indicate that, although the GCS remains a strong initial prognostic indicator, it may be insufficient when used in isolation. Incorporating pupillary response and CT-based anatomical indicators results in a more comprehensive and systematic risk estimation framework. Importantly, all variables included in the model are available at the time of admission, enabling early risk assessment without the need for advanced imaging or additional laboratory markers. From a practical perspective, the proposed model may support structured early risk estimation by combining neurological examination with readily available CT parameters during the acute phase of care. The model should therefore be interpreted as a framework for early risk estimation rather than a standalone clinical decision tool. Recent developments in computational imaging and data-driven approaches have further highlighted the potential role of structured radiological features as inputs for emerging analytical models, and such approaches may provide a basis for future integration of CT-based parameters into more advanced predictive frameworks [26].

One of the strengths of this study lies in the systematic integration of clinical neurological assessment and CT-based radiological parameters within a single model. The anatomical burden score was explicitly defined, providing a structured and reproducible representation of injury extent. The assessment of both discrimination and calibration, together with internal validation using bootstrap resampling, supports the internal robustness of the findings. Moreover, radiological evaluations were reassessed by a second neurosurgeon blinded to clinical outcomes, and interobserver agreement for radiological variables was in the good-to-excellent range, reinforcing measurement reliability.

Nevertheless, this study is retrospective and was conducted at a single centre. The anatomical burden score represents a pragmatic composite indicator rather than a previously validated weighted prognostic index, and its performance should therefore be interpreted in the context of the present dataset. The model has not undergone external validation in an independent cohort, and therefore, its performance in other settings remains to be determined. In addition, the extended study period (2005–2025) may introduce temporal heterogeneity related to changes in diagnostic and treatment practices over time. Long-term functional outcomes were not analysed, and complications were not incorporated into the prognostic model. Furthermore, exclusion of patients who died before neurosurgical assessment may have resulted in a degree of selection bias toward patients with less severe initial presentations. In addition, interobserver agreement analysis was performed on a subsample, which may limit generalisability, particularly because the findings may not accurately reflect the broader population’s variability in outcomes. Furthermore, the highest anatomical burden score category contained only a small number of patients, which limits precision at the upper end of the score distribution. However, as the anatomical burden score was analysed as a continuous variable, the impact of this sparse category on the model is limited. Future multicentre and prospective studies may help validate the anatomical burden score across diverse populations, and the incorporation of long-term functional outcomes could further refine early risk stratification strategies.

## 5. Conclusions

In adult CGSIs, a low GCS score at admission, bilateral non-reactive pupils, an increased anatomical burden score, and increased midline shift are associated with a higher risk of in-hospital mortality. Integrating clinical findings with CT-based anatomical indicators may support a more structured early estimation of mortality risk, although further external validation is required before broader clinical application.

## Figures and Tables

**Table 1:** Baseline demographic, clinical, and radiological characteristics according to in-hospital mortality.

Variable	Survivors (*n* = 62)	Non-Survivors (*n* = 81)	*p* Value
Age, years *	34 (22–49)	42 (28–57)	0.018
Male sex, *n* (%)	50 (80.6)	68 (84.0)	0.612
GCS ≤ 8, *n* (%)	18 (29.0)	56 (69.1)	<0.001
Bilateral non-reactive pupils, *n* (%)	4 (6.5)	28 (34.6)	<0.001
Anatomical burden score *	1 (0–2)	2 (1–3)	<0.001
Midline shift (mm) *	2 (0–4)	6 (3–9)	<0.001
Posterior fossa involvement, *n* (%)	6 (9.7)	18 (22.2)	0.048

* Continuous variables are presented as median (IQR). Categorical variables are presented as *n* (%). The Mann–Whitney U test was performed for continuous variables, and the chi-square or Fisher’s exact test was performed for categorical variables, as appropriate. GCS = Glasgow Coma Scale.

**Table 2:** In-hospital mortality according to the anatomical burden score.

Anatomical Burden Score	*n*	Deaths (*n*)	Mortality (%)
0	44	13	29.5
1	52	34	65.4
2	37	25	67.6
3	9	8	88.9
4	1	1	100.0

Note: Values are presented as *n* (%). In-hospital mortality across anatomical burden score categories was compared using Fisher’s exact test; linear trend across categories was evaluated using the Cochran–Armitage test. The anatomical burden score (0–4) was calculated by assigning one point each for multilobar involvement, cross-midline trajectory, IVH, and deep structure involvement. Prognostic modelling was performed using the anatomical burden score as a continuous variable.

**Table 3:** Therapeutic interventions and associated clinical outcomes.

Treatment Category	*n* (%)	Deaths, *n* (%)	Complications, *n* (%)	Median GCS (IQR)
Surgical intervention (total)	107 (74.8%)	64 (59.8%)	16 (15.0%)	6 (4–8)
Decompressive craniectomy and haematoma evacuation	63 (44.1%)	45 (71.4%)	11 (17.5%)	4 (3–7)
Wound debridement and foreign body removal	44 (30.7%)	19 (43.2%)	5 (11.4%)	8 (6–10)
Conservative treatment	36 (25.2%)	17 (47.2%)	4 (11.1%)	9 (7–12)

Note: Values are presented as *n* (%) or median (IQR). Comparisons between treatment groups are descriptive; no causal inference was intended due to potential indication bias and differences in neurological severity at admission. GCS: Glasgow Coma Scale; IQR: interquartile range.

**Table 4:** Multivariable Logistic Regression Models.

Variable	Model 1 OR (95% CI)	*p*-Value	Model 2 OR (95% CI)	*p*-Value
Midline shift (per mm)	1.13 (1.07–1.20)	<0.001	1.12 (1.06–1.19)	<0.001
Anatomical burden score (per point)	1.94 (1.35–2.78)	<0.001	1.88 (1.30–2.70)	0.001
Bilateral non-reactive pupil	4.38 (1.62–11.84)	0.003	4.05 (1.48–11.10)	0.006
GCS ≤ 8	6.12 (2.85–13.15)	<0.001	5.82 (2.60–13.02)	<0.001
Treatment (surgical vs. conservative)	–	–	0.89 (0.42–1.87)	0.76

Note: Continuous variables were modelled per unit increase. Odds ratios were derived from multivariable logistic regression models. Both the primary admission-based model and the extended model including treatment variables are presented. Model stability was confirmed using Firth’s penalised logistic regression.

## Data Availability

The data presented in this study are available upon reasonable request from the corresponding author. The data are not publicly available due to patient privacy restrictions and institutional policies regarding the confidentiality of medical records. The Institutional Review Board of Adana City Training and Research Hospital may approve the sharing of anonymised datasets with qualified researchers.

## Abbreviations

AUC	Area under the curve
CGSI	Cranial gunshot injury
CI	Confidence interval
CSF	Cerebrospinal fluid
CT	Computed tomography
GCS	Glasgow Coma Scale
GOS	Glasgow Outcome Scale
ICC	Intraclass correlation coefficient
ICP	Intracranial pressure
IQR	Interquartile range
IVH	Intraventricular haemorrhage
OR	Odds ratio
ROC	Receiver operating characteristic
SD	Standard deviation
TBI	Traumatic brain injury
VIF	Variance inflation factor

## References

[1] Lilford R.D., Hossain I., Dahlberg M., Wahlgren C.-M., Bellander B.-M., Rostami A., Günther M., Bartek J., Rostami E. (2024). Increased incidence and mortality of civilian penetrating traumatic brain injury in Sweden: A single-center registry-based study. World Neurosurg..

[2] Waltzman D., Sarmiento K., Daugherty J., Lumba-Brown A., Klevens J., Miller G.F. (2023). Firearm-related traumatic brain injury homicides in the United States, 2000–2019. Neurosurgery.

[3] Maragkos G.A., Papavassiliou E., Stippler M., Filippidis A.S. (2018). Civilian gunshot wounds to the head: Prognostic factors affecting mortality: Meta-analysis of 1774 patients. J. Neurotrauma.

[4] Stefanopoulos P.K., Breglia G.A., Bissias C., Nikita A.S., Papageorgiou C., Tsiatis N.E., Serafetinides E., Gyftokostas D.A., Aloizos S., Mikros G. (2025). Firearm injuries: A review of wound ballistics and related emergency management considerations. Emerg. Care Med..

[5] Aarabi B., Tofighi B., Kufera J.A., Hadley J., Ahn E.S., Cooper C., Malik J.M., Naff N.J., Chang L., Radley M. (2014). Predictors of outcome in civilian gunshot wounds to the head. J. Neurosurg..

[6] Loggini A., Vasenina V.I., Mansour A., Das P., Horowitz P.M., Goldenberg F.D., Kramer C., Lazaridis C. (2020). Management of civilians with penetrating brain injury: A systematic review. J. Crit. Care.

[7] Alexopoulos G., Quadri N., Khan M., Bazai H., Pico C.F., Fraser C., Kulkarni N., Kemp J., Coppens J., Bucholz R. (2021). Ballistic lobar trajectory outcomes in civilian firearm penetrating brain injury. J. Neurosurg..

[8] Sorek S., Miller A., Mathew V., Moawad S., Rahme R. (2023). Gunshot wound to the posterior fossa with a transcerebellar retromesencepahlic bullet path, transient mutism, and unexpected functional recovery: The pivotal, energy-absorbing function of the petrous bone and tentorial leaflet. Cureus.

[9] Javeed F., Abbas A., Rehman L., Rizvi S.R.K., Afzal A., Aziz H.F. (2020). Outcome of cranial firearm injuries in civilian population based on a novel classification system. Surg. Neurol. Int..

[10] Carney N., Totten A.M., O’Reilly C., Ullman J.S., Hawryluk G.W., Bell M.J., Bratton S.L., Chesnut R., Harris O.A., Kissoon N. (2017). Guidelines for the management of severe traumatic brain injury, fourth edition. Neurosurgery.

[11] Kochanek P.M., Tasker R.C., Carney N., Totten A.M., Adelson P.D., Selden N.R., Davis-O’reilly C., Hart E.L., Bell M.J., Bratton S.L. (2019). Guidelines for the management of pediatric severe traumatic brain injury, third edition: Update of the Brain Trauma Foundation guidelines. Pediatr. Crit. Care Med..

[12] Maas A.I.R., Menon D.K., Adelson P.D., Andelic N., Bell M.J., Belli A., Bragge P., Brazinova A., Büki A., Chesnut R.M. (2017). Traumatic brain injury: Integrated approaches to improve prevention, clinical care, and research. Lancet Neurol..

[13] Prabhakar H., Tripathy S., Gupta N., Singhal V., Mahajan C., Kapoor I., Wanchoo J., Kalaivani M. (2021). Consensus statement on analgo-sedation in neurocritical care and review of literature. Indian J. Crit. Care Med..

[14] Ganga A., Leary O.P., Sastry R.A., Asaad W.F., Svokos K.A., Oyelese A.A., Mermel L.A. (2023). Antibiotic prophylaxis in penetrating traumatic brain injury: Analysis of a single-center series and systematic review of the literature. Acta Neurochir..

[15] Robba C., Poole D., McNett M., Asehnoune K., Bösel J., Bruder N., Chieregato A., Cinotti R., Duranteau J., Einav S. (2020). Mechanical ventilation in patients with acute brain injury: Recommendations of the European Society of Intensive Care Medicine consensus. Intensive Care Med..

[16] Oktay K., Mammadov M., Alnageeb A., Alcan H., Pektaş U., Ozsoy K., Cetinalp N., Erman T. (2025). Evaluation of prognostic factors in patients with cranial gunshot wounds. Niger. J. Clin. Pract..

[17] Lee S.-J., Chen Y.-L., Wu T.-H., Liu C.-Y., Wang C.-H., Tsai C.-H., Chung J.-Y., Yiang G.-T., Wu M.-Y. (2025). Comparison of Glasgow coma scale, motor component, eye component, and simplified motor scale for predicting trauma outcomes: A 13-year multicenter retrospective cohort study. BMC Emerg. Med..

[18] Abojarad B., Elhissi A.J.H., Aldabbour B. (2025). War-related traumatic brain injury in Gaza: A multi-center prospective analysis of patterns and outcomes. Scand. J. Trauma. Resusc. Emerg. Med..

[19] Nyam T.-T.E., Tu K.-C., Kuo Y.-H., Wang C.-C., Liu C.-F., Liao J.-C., Kuo C.-L. (2025). Age and pupil size: Key predictors of mortality in traumatic brain injury patients with GCS 3. Front. Neurol..

[20] Jahns F.-P., Miroz J.P., Messerer M., Daniel R.T., Taccone F.S., Eckert P., Oddo M. (2019). Quantitative pupillometry for the monitoring of intracranial hypertension in patients with severe traumatic brain injury. Crit. Care.

[21] DeCuypere M., Muhlbauer M.S., Boop F.A., Klimo P. (2016). Pediatric intracranial gunshot wounds: The Memphis experience. J. Neurosurg. Pediatr..

[22] Emami P., Czorlich P., Fritzsche F.S., Westphal M., Rueger J.M., Lefering R., Hoffmann M. (2017). Impact of Glasgow Coma Scale score and pupil parameters on mortality rate and outcome in pediatric and adult severe traumatic brain injury: A retrospective multicenter cohort study. J. Neurosurg..

[23] Marshall L.F., Marshall S.B., Klauber M.R., Van Berkum Clark M., Eisenberg H., Jane J.A., Luerssen T.G., Marmarou A., Foulkes M.A. (1991). A new classification of head injury based on computerized tomography. J. Neurosurg..

[24] Hukkelhoven C.W.P., Steyerberg E.W., Habbema J.D.F., Farace E., Marmarou A., Murray G.D., Marshall L.F., Maas A.I.R. (2005). Predicting outcome after traumatic brain injury: Development and validation of a prognostic score based on admission characteristics. J. Neurotrauma.

[25] Mohamed H., Aljondi R., Alhailiy A., Mahmoud M.Z. (2021). Detection of midline shift from CT scans to predict outcome in patients with head injuries. Int. J. Biomed..

[26] Sessa F., Chisari M., Esposito M., Guardo E., Di Mauro L., Salerno M., Pomara C. (2025). Advancing Diagnostic Tools in Forensic Science: The Role of Artificial Intelligence in Gunshot Wound Investigation—A Systematic Review. Forensic Sci..

